# Incorporation of a Stress Reducing Mobile App in the Care of Patients With Type 2 Diabetes: A Prospective Study

**DOI:** 10.2196/mhealth.7408

**Published:** 2017-05-29

**Authors:** Maya Munster-Segev, Oren Fuerst, Steven A Kaplan, Avivit Cahn

**Affiliations:** ^1^ Diabetes Unit Department of Internal Medicine Hadassah Hebrew University Hospital Jerusalem Israel; ^2^ Eco-Fusion Herzliya Israel; ^3^ Icahn School of Medicine and Director of the Men's Wellness Program Mount Sinai New York, NY United States; ^4^ Endocrinology and Metabolism Unit Department of Internal Medicine Hadassah Hebrew University Hospital Jerusalem Israel

**Keywords:** diabetes mellitus, type 2, biofeedback, physiological stress response, mobile health, telemedicine

## Abstract

**Background:**

Severe and sustained emotional stress creates a physiological burden through increased sympathetic activity and higher energy demand. This may lead to increased oxidative stress and development of the metabolic syndrome. Emotional stress has been shown to contribute to the onset, progression, and control of type 2 diabetes (T2D). Stress management and biofeedback assisted relaxation have been shown to improve glycemic control. Use of a mobile app for stress management may enhance the scalability of such an approach.

**Objective:**

The aim of this study was to assess the effect of using a mobile app of biofeedback-assisted relaxation on weight, blood pressure (BP), and glycemic measures of patients with T2D.

**Methods:**

Adult patients with T2D and inadequate glycemic control (hemoglobin A1c [HbA1c]>7.5%) were recruited from the outpatient diabetes clinic. Baseline weight, BP, HbA1c, fasting plasma glucose (FPG), triglycerides (TG), and 7-point self-monitoring of blood glucose were measured. Patients were provided with a stress reducing biofeedback mobile app and instructed to use it 3 times a day. The mobile app—Serenita—is an interactive relaxation app based on acquiring a photoplethysmography signal from the mobile phone’s camera lens, where the user places his finger. The app collects information regarding the user’s blood flow, heart rate, and heart rate variability and provides real-time feedback and individualized breathing instructions in order to modulate the stress level. All clinical and biochemical measures were repeated at 8 and 16 weeks of the study. The primary outcome was changes in measures at 8 weeks.

**Results:**

Seven patients completed 8 weeks of the study and 4 completed 16 weeks. At week 8, weight dropped by an average of 4.0 Kg (SD 4.3), systolic BP by 8.6 mmHg (SD 18.6), HbA1c by 1.3% (SD 1.6), FPG by 4.3 mmol/l (4.2), and serum TG were unchanged.

**Conclusions:**

Stress reduction using a mobile app based on biofeedback may improve glycemic control, weight, and BP.

## Introduction

Type 2 diabetes (T2D) is a chronic disease which has reached epidemic proportions worldwide. Recent data show a total of 415 million patients in the world suffering from diabetes with an expected rise to 642 million by 2040 [1]. Factors contributing to this alarming rise in the prevalence of diabetes include obesity compounded by a sedentary lifestyle, increasing life-expectancy, urbanization, reduced physical activity, increased sugar consumption, and low fruit and vegetable intake [1].

Severe and sustained emotional stress creates a physiological burden through increased sympathetic activity and higher energy demand. This may lead to increased oxidative stress and development of conditions such as the metabolic syndrome, accelerated aging, and cardiovascular disease [2]. Emotional stress has been shown to be a significant accelerator to the development and progression of chronic diseases in general, and T2D in particular [3-5]. Furthermore, individuals experiencing increased levels of stress may find it more difficult to manage their chronic condition, often leading to its exacerbation and resulting in increased stress.

Mind-body interventions facilitate autonomic flexibility, enhance self-regulation, and induce a “relaxation response” which is characterized by parasympathetic dominance, reduced sympathetic activation, and increased heart rate variability. Yoga includes a wide range of mind-body practices including postures, breathing, meditation, and relaxation practices that are reported to counteract physiological stress. Compared with nonyoga practitioners, regular yoga practitioners are reported to have lower heart rate, breath rate, blood pressure (BP), metabolic rate, and higher heart rate variability. Regular yoga practice inspires a sense of psychological and physiological equilibrium that is different from rest, physical relaxation, and sleep [2]. Yoga has been found to be beneficial in reducing oxidative stress in T2D [6].

Incorporation of stress management [7,8] as well as biofeedback-assisted relaxation [9] in the care of diabetes to facilitate increased patient compliance and improved glycemic control has been previously described.

Serenita is a mobile app that engages the body’s natural relaxation response by using a personalized breathing exercise. In this study, we assessed the effect of incorporating this novel stress management mobile app in the care of patients with diabetes.

## Methods

### Study Oversight

This was a single center study conducted in in the diabetes unit of Hadassah Hebrew University Medical Center. The study was approved by the Institutional Ethics Board. All subjects provided signed informed consent (NCT 02691273).

### Study Population

The study included patients aged 18 years or above with T2D duration of at least one year who were willing to comply with the study protocol. Patients’ hemoglobin A1c (HbA1c) was >7.5%, and they had not attained their glycemic target as assessed by the investigators. Access to a mobile phone—either Android version 4 and up or iPhone 4 and up—with iOS 7 and up was mandatory.

Patients with type 1 diabetes, pregnant women, or those suffering from an acute disease or a chronic significantly decompensated disorder were excluded from the trial.

### Study Outcomes

This was a single-arm pilot study designed to assess the impact of using a stress reducing mobile app on the glycemic control, weight, and BP of patients with T2D. The main outcomes were changes in HbA1c, triglycerides (TG), fasting plasma glucose (FPG), 7-point daily glycemic profile, weight, and blood from baseline to week 8. Compliance with the use of the mobile app was to be assessed as well. Patients additionally completed a quality of life questionnaire (SF-12) [10], as well as questionnaires regarding multiple aspects of daily life including sleeping hours, mood, exercise, and hunger at baseline and at week 8. Measures were repeated at week 16.

### Study Conduct

Patients attending the outpatient diabetes clinic and willing to participate were screened for the trial. Eligible patients were enrolled in the study.

At baseline, medical history was obtained and a physical exam was conducted. Vital signs, weight, BP, and blood test were taken. The patients were requested to complete a 7-point glucose profile in the 2 days before baseline visit. Dietary counseling in accordance with the American Diabetes Association (ADA) clinical nutrition guidelines was delivered by a registered dietician [5]. Each patient received detailed instructions on how to install and operate the mobile app. Patients were requested to fill out the SF-12 and the questionnaire regarding daily life routine.

The patients received subsequent weekly phone calls to assess their compliance with the mobile app, dietary recommendations, and any further impending issues for 8 weeks, and then monthly calls till week 16. At 8 and 16 weeks after baseline, patients were invited to the clinic where anthropometric measures, blood tests, and 7-point glucose measurements were reassessed, in addition to the reinforcement of dietary guidelines and completion of the questionnaires.

### Description of the Mobile App

The mobile app—Serenita—is an interactive relaxation app based on acquiring a photoplethysmography signal from the mobile phone’s camera lens, where the user places his finger [11]. Information related to the user’s blood flow, heart rate, and heart rate variability is extracted. These signals are then filtered and processed using a large array of algorithms and machine learning to assess the user’s physiological stress level and subsequently recommend real-time stress reducing interactive breathing patterns.

**Table 1:** Baseline and follow-up weight and blood pressure.

Patient #	Visit	Weight (kg)	Change from baseline	Systolic BP^a^ (mmHg)	Change from baseline	Diastolic BP (mmHg)	Change from baseline
1	Baseline	79		156		80	
	Week 8	76.6	−2.4				
	Week 16	77.5	−1.5	148	−8	81	1
3	Baseline	110.2		126		84	
	Week 8	103.2	−7				
	Week 16	100	−10.2	111	−15	69	−15
4	Baseline	105.6		140		83	
	Week 8	102.1	−3.5	151	11	84	1
	Week 16	101.4	−4.2				
8	Baseline	122.2		148		86	
	Week 8	110	−12.2	118	−30	75	−11
	Week 16	109.5	−12.7				
11	Baseline	74.2		137		90	
	Week 8	74.4	0.2	122	−15	79	−11
	Week 16						
12	Baseline	89.1		133		74	
	Week 8	86.7	−2.4	113	−20	71	−3
	Week 16						
14	Baseline	62		121		88	
	Week 8	61.5	−0.5	131	10	84	−4
	Week 16						
Average (SD)^b^ (week 8)	N	7		5		5	
	Baseline	91.8 (21.7)		135.6 (9.8)		84.2 (6.4)	
	Week 8	87.8 (17.9)	−4.0 (4.3)	127.0 (15.2)	−8.6 (18.6)	78.7 (5.8)	−5.5 (5.1)
Average (SD) (week 16)	N	4		2		2	
	Baseline	104.3 (18.2)		141.0 (21.2)		82.2 (3.1)	
	Week 16	97.1 (13.7)	−7.2 (5.2)	129.3 (26.4)	−11.7 (5.2)	75.0 (8.5)	−7.2 (11.5)

^a^BP: blood pressure.

^b^SD: standard deviation.

**Figure 1:**
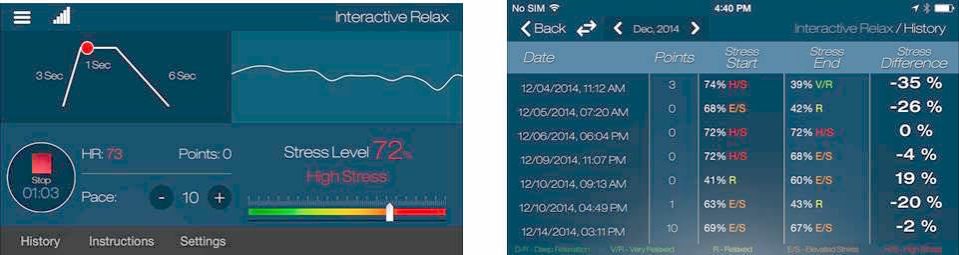
Screen cap from the mobile app.

Serenita encourages deep diaphragmatic breathing, often referred to as yogic breathing, as well as coherence breathing—breathing synchronized to the heart rate. Additionally, the app offers the user real-time feedback on the effect of their breathing pattern on additional physiological parameters such as heart rate and heart rate variability and accordingly reflects to the individual their current stress level. The mobile app optimizes the pattern of breathing so that the user can assess his or her physiological stress level and obtain a personalized breathing pattern to rapidly reduce the level of stress and increase focus. Figure 1 shows screen caps from the app.

Patients were instructed to use the mobile app 3 times a day for 10 min each session.

### Statistical Analyses

This study was designed as a pilot study, and sample size determination was not planned to meet any specific significance and power requirements. Descriptive analyses were conducted for all measurements in the study.

## Results

Fifteen patients were screened for the study and 9 patients were enrolled. Causes for screen failure included low HbA1c (3), incompatible mobile phone (1), lack of home WiFi connection (1), and personal reasons (1). Of the 9 patients enrolled, 7 completed the study. One was unable to operate the app, as the heart rate signal was not adequately transmitted (possibly due to thick skin in his fingers), and one was lost to follow-up after the baseline visit. Seven patients attended the visit at week 8 and 4 attended the visit at week 16. The average age was 55 (SD 11.6), 71% (5/7) were male, diabetes duration was 11 years (SD 7.3), and 71% (5/7) had been using insulin. Baseline, visit 8, and visit 16 weight, BP, HbA1c, FPG, and TG are shown in Tables 1 and 2. At week 8, weight dropped by an average of 4.0

Kg (SD 4.3) from a baseline of 91.8 Kg (SD 21.7) (n=7); systolic BP dropped in average by 8.6 mmHg (SD 18.6) (n=5); HbA1c was reduced by an average of 1.3% (SD 1.6) from 9.0% (SD 0.7) at baseline; FPG was reduced by 4.3 mmol/l (SD 4.2) (mean 77.4 mg/dl, SD 75.6), and serum TG were unchanged (n=6). Baseline medications remained unchanged throughout the trial, except for a 30% increase in insulin dose of patient #11.

**Figure 2:**
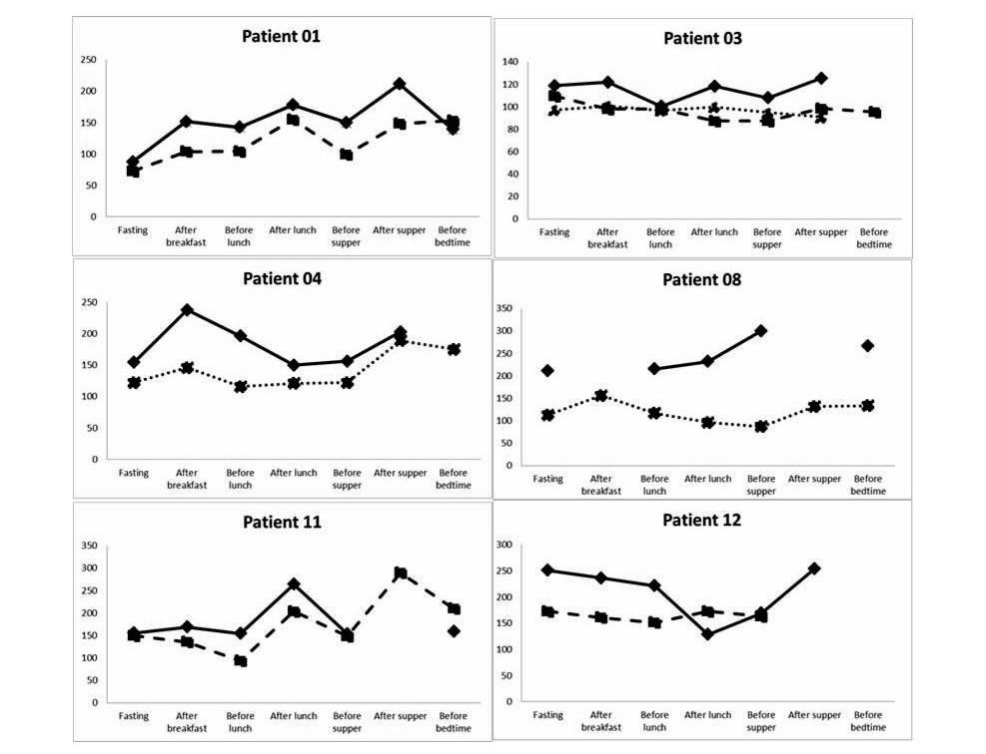
Self-monitoring blood glucose levels of the individual patients. Legend—self-monitoring of blood glucose (SMBG) levels. Solid lines—baseline, dashed line—week 8 visit, dotted line—week 16 visit. Patient 14 did not complete SMBG.

**Table 2:** Baseline and follow-up biochemistry analyses.

Patient #	Visit	HbA1c^a^ (%)	Change from baseline	FPG^b^ (mmol/l)	Change from baseline	TG^c^ (mmol/l)	Change from baseline
1	Baseline	7.6		8.1		1.5	
	Week 8	6.3	−1.3	5.5	−2.6	1.6	0.1
	Week 16	6.2	−1.4	3.5	−4.6	1.8	0.3
3	Baseline	9.7		18.5		1.7	
	Week 8	5.2	−4.5	5.6	−12.9	1.2	−0.5
	Week 16	5.1	−4.6	4.6	−13.9	1.5	−0.2
4	Baseline	9.1		10.7		2.3	
	Week 8	8.2	−0.9	7.6	−3.1	1.8	−0.5
	Week 16						
8	Baseline	10.4		8.8		3	
	Week 8						
	Week 16	6.1	−4.3	3.8	−5	1.7	−1.3
11	Baseline	9.4		5.7		0.9	
	Week 8	10.2	0.8	5.2	−0.5	0.9	0
	Week 16						
12	Baseline	9.2		13.5		1.6	
	Week 8	8.2	−1	12.3	−1.2	2.5	0.9
	Week 16						
14	Baseline	8.8		8		2	
	Week 8	7.7	−1.1	2.7	−5.3	2.2	0.2
	Week 16						
Average (SD) (week 8)	n	6		6		6	
	Baseline	9.0 (0.7)		10.8 (4.6)		1.7 (0.5)	
	Week 8	7.6 (1.6)	−1.3 (1.6)	6.5 (3.0)	−4.3 (4.2)	1.7 (0.6)	0.0 (0.5)
Average (SD) (week 16)	n	3		3		3	
	Baseline	9.2 (1.5)		11.8 (5.8)		2.1 (0.8)	
	Week 16	5.8 (0.6)	−3.4 (1.6)	4.0 (0.6)	−7.8 (5.3)	1.7 (0.2)	−0.4 (0.8)

^a^HbA1c: hemoglobin A1c.

^b^FPG: fasting plasma glucose.

^c^TG: triglycerides.

^d^SD: standard deviation.

Seven-point glucose measurements at baseline and at visits 8 and 16 are shown in Figure 2. Patients’ SF-12 and daily life questionnaires were similar between baseline and subsequent visits (Multimedia Appendix 1). Data regarding the cumulative time the mobile app was used was not available due to a technical malfunction, yet, most patients reported it was easy for them to adhere to the program (Multimedia Appendix 1).

## Discussion

### Principal Findings

Our study demonstrates an improvement in weight, BP, and glycemic parameters of patients provided with a stress reducing mobile app, in addition to standard of care.

The use of biofeedback-assisted relaxation in patients with T2D has been previously described. Mcginnis et al randomized 39 patients to a regimen of either 10 sessions of biofeedback and relaxation or 3 sessions of education. Biofeedback and relaxation were associated with significant decreases in average blood glucose, HbA1c, and muscle tension compared with the control group [9].

Jyotsna et al randomized 120 patients to either standard diabetes care or to a yogic breathing program for 6 months. At 6 months, quality of life and postprandial plasma glucose signiﬁcantly improved in the group practicing yoga compared with baseline, but there was no signiﬁcant improvement in the FPG and in HbA1c [12]. Biofeedback has also been shown to reduce food craving [13] as well as BP [14,15].

There may be multiple mechanisms whereby biofeedback-assisted relaxation or yogic breathing may improve glycemic control. Activation of the sympathetic system during stress increases cortisol levels and insulin resistance. Biofeedback has been shown to decrease multiple aspects of the chronic physiological stress response including muscle tension, peripheral vasoconstriction, heart rate, cortisol, and cathecolamines. Biofeedback may also carry psychological effects such as an improved sense of control and better problem solving [8]. Diabetes control is largely dependent on the compliance of the patient with the drug and dietary regimen prescribed and patient empowerment is an inseparable aspect of diabetes care [16]. Improved problem solving and sense of control as well as reduction of depression, anxiety, and improved sleep patterns secondary to biofeedback may also contribute to improved glycemic control [14].

### Strength and Limitations

The benefit of our approach of incorporating a mobile app stress reducing technique is its scalability. The app can be delivered to thousands of patients without incurring the cost of face-to-face classes of yogic breathing or biofeedback. In the current era where the use of mobile devices is increasing rapidly, incorporating mobile apps in the motivational programs for patients with diabetes may be effectively cost reducing.

Several limitations to our report must be recognized. The study was designed as a pilot study, and the number of patients completing the trial was small. Additionally, the trial included both the use of the mobile app as well as dietary consultation and weekly phone contact. Although patients had long-standing diabetes and were already well educated in the concepts of healthy diabetes nutrition, there are still well-recognized benefits of a “refresher” course in medical nutrition therapy, particularly when paralleled by weekly motivational calls. Finally, due to technical reasons, data regarding the extent of use of the app was not available, therefore, it is not possible to assess whether the extent of clinical benefit correlated with the extent of app usage.

Nevertheless, the results of the study as well as the positive feedback received from our patients encourage expansion of the study to a larger-scale randomized trial, wherein patients are randomized to standard of care alone or with the addition of the mobile app.

## Appendix

Multimedia Appendix 1 Patient questionnaires.

## Abbreviations

BP: blood pressure
FPG: fasting plasma glucose
HbA1c: hemoglobin A1c
SD: standard deviation
T2D: type 2 diabetes
TG: triglycerides
SMBG: self-monitoring of blood glucose
