# Supplementary material for: Incorporation of a Stress Reducing Mobile App in the Care of Patients With Type 2 Diabetes: A Prospective Study
Source: JMIR Mhealth Uhealth. 2017 May 29;5(5):e75. doi: 10.2196/mhealth.7408 (PMC5468539; doi:10.2196/mhealth.7408)
Supplement: Multimedia Appendix 1 [file mhealth_v5i5e75_app1.pdf]

		SF-12 – Question number											
**Patient #**	**Visit**	**1**	**2**	**3**	**4**	**5**	**6**	**7**	**8**	**9**	**10**	**11**	**12**
**1**	Baseline	3	3	2	2	2	2	2	1	2	3	6	5
	Week 8	3	3	2	2	2	2	2	1	2	2	6	5
	Week 16	3	3	2	2	2	2	2	2	4	4	4	5
**3**	Baseline	4	3	3	1	2	2	2	3	5	4	4	5
	Week 8	3	2	2	2	2	2	2	2	4	4	4	3
	Week 16	4	3	2	2	2	2	2	1	4	4	5	5
**4**	Baseline	4	2	2	1	1	1	2	3	4	4	5	4
	Week 8	4	3	3	1	1	1	1	3	5	5	4	5
	Week 16	4	2	3	1	1	1	1	2	4	5	3	4
**8**	Baseline	5	2	1	1	1	1	1	4	5	5	3	2
	Week 8												
	Week 16	4	2	1	1	1	1	1	4	5	6	2	1
**11**	Baseline	2	3	3	2	2	2	2	1	2	2	5	5
	Week 8	2	3	3	2	2	2	2	1	3	3	6	5
	Week 16												
**12**	Baseline	3	1	1	2	2	2	2	1	6	1	3	5
	Week 8	4	3	1	1	1	2	2	2	4	2	4	3
	Week 16												
**14**	Baseline	4	3	3	2	2	2	2	1	2	2	6	5
	Week 8	3	3	3	2	2	2	2	1	2	2	6	5
	Week 16												

SF-12

1. In general you would say your health is
   1. Excellent
   2. Very good
   3. Good
   4. Fair
   5. Poor

The following items are about activities you might do during a typical day. Does your health now limit you in these activities? If so, how much?

2. Moderate activities such as moving a table, pushing a vacuum cleaner, bowling or playing golf
   1. Yes. Limited a lot
   2. Yes, limited a little
   3. No, not limited at all

3. Climbing several flights of stairs
   1. Yes. Limited a lot
   2. Yes, limited a little
   3. No, not limited at all

During the past 4 weeks, have you had any of the following problems with your work or other regular daily activities as a result of your physical health?

4. Accomplished less than you would like.
   1. Yes
   2. No
5. Were limited in the kind of work or other activities.
   1. Yes
   2. No

During the past 4 weeks, have you had any of the following problems with your work or other regular daily activities as a result of any emotional problems (such as feeling depressed or anxious)?

6. Accomplished less than you would like.
   1. Yes
   2. No
7. Did work or activities less carefully than usual.
   1. Yes
   2. No
8. During the past 4 weeks, how much did pain interfere with your normal work (including work outside the home and housework)?
   1. Not at all
   2. A little bit
   3. Moderately
   4. Quite a bit
   5. Extremely

These questions are about how you have been feeling during the past 4 weeks. For each question, please give the one answer that comes closest to the way you have been feeling. How much of the time during the past 4 weeks…

9. Have you felt calm & peaceful?
   1. All of the time
   2. Most of the time
   3. A good bit of the time
   4. Some of the time
   5. A little of the time
   6. None of the time
10. Did you have a lot of energy?
    1. All of the time
    2. Most of the time
    3. A good bit of the time
    4. Some of the time
    5. A little of the time
    6. None of the time
11. Have you felt down-hearted and blue?
    1. All of the time
    2. Most of the time
    3. A good bit of the time
    4. Some of the time

5. A little of the time
6. None of the time

12. During the past 4 weeks, how much of the time has your physical health or emotional problems interfered with your social activities (like visiting friends, relatives, etc.)?
    1. All of the time
    2. Most of the time
    3. Some of the time
    4. A little of the time
    5. None of the time

		Daily life Question number								
**Patient #**	**Visit**	**1**	**2**	**3**	**4**	**5**	**6**	**7**	**8**	**9**
**1**	Baseline		6	3	2	30	8-9	1	3	
	Week 8									
	Week 16	Tired	6	3	5	30	>10	3	3	1
**3**	Baseline	Great	7	1	0	0	5<	1	3	
	Week 8									
	Week 16	Tired	5	1	3	60	6-7	2	5	1
**4**	Baseline	Alert	4	2	3	30	5<	3	3	
	Week 8									
	Week 16	OK	6	3	>5	50-60	8-9	2	5	4
**8**	Baseline									
	Week 8	Tired	5	4	2	10-15	8-9	3	2	
	Week 16	Weak	5	3	3	15	8-9	3	3	2
**11**	Baseline									
	Week 8	Very good	6	1	2	40	5<	2	3	4
	Week 16									
**12**	Baseline	Normal	4.5	3	0	0	5<	5	3	
	Week 8	Average	5.5	3	0	0	5<	5	3	2
	Week 16									
**14**	Baseline	Good	5	2	2	60	>10	2	1	
	Week 8	Excellent	6	1	2	40	6-7	1	3	1
	Week 16									

Daily life questionnaire*

1. How did you feel this morning when you woke up?
2. How many hours did you sleep last night?
3. How is your mood this morning (1=excellent, 5=terrible)
4. How many times did you exercise this week?
5. How long was each session (minutes)?
6. How many glasses of water do you drink during the day?
7. How frequently did you experience stress during the day? (1=never, 5=frequently)
8. Have you felt hungry between the meals? (1=very much, 5=not at all)
9. How difficult was it for you to adhere to the program? (1=very easy, 5=very difficult)

*Excluding free text questions
